# Developing a “Sepsis Never Event” Measure for Our Hospital-wide Improvement Initiative

**DOI:** 10.1097/pq9.0000000000000636

**Published:** 2023-02-23

**Authors:** Elise Rolison, Carter Smith, Beth Wathen, Halden Scott, Sarah Nickels, Justin M. Lockwood

**Affiliations:** From the * Clinical Effectiveness Team, Children’s Hospital Colorado; † Pediatric Intensive Care Unit, Children’s Hospital Colorado; ‡ Department of Pediatrics, Section of Emergency Medicine, University of Colorado School of Medicine; § Department of Pediatrics, Section of Hospital Medicine, University of Colorado School of Medicine.

## Background:

Historical measure definitions for our sepsis initiative captured intent-to-treat without connection to final diagnosis or patient outcomes.

## Objectives:

To develop a novel sepsis outcome measure capturing confirmed sepsis-related patient harm including a reliable process to identify cases fulfilling its criteria.

## Methods:

**Fig. 1.**
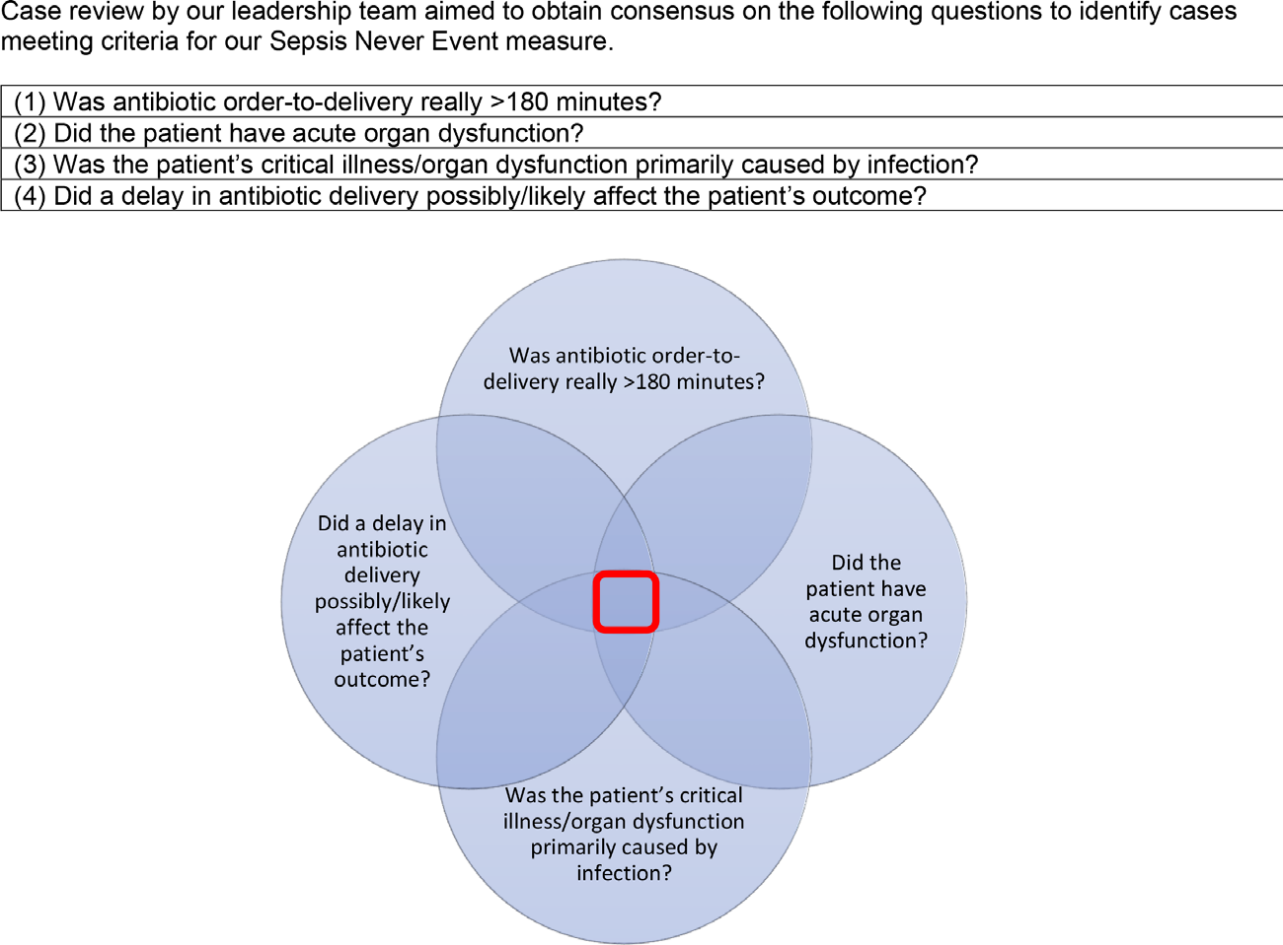
Case review by our leadership team aimed to obtain consensus on the following questions to identify cases meeting criteria for our Sepsis Never Event measure.

**Fig. 2.**
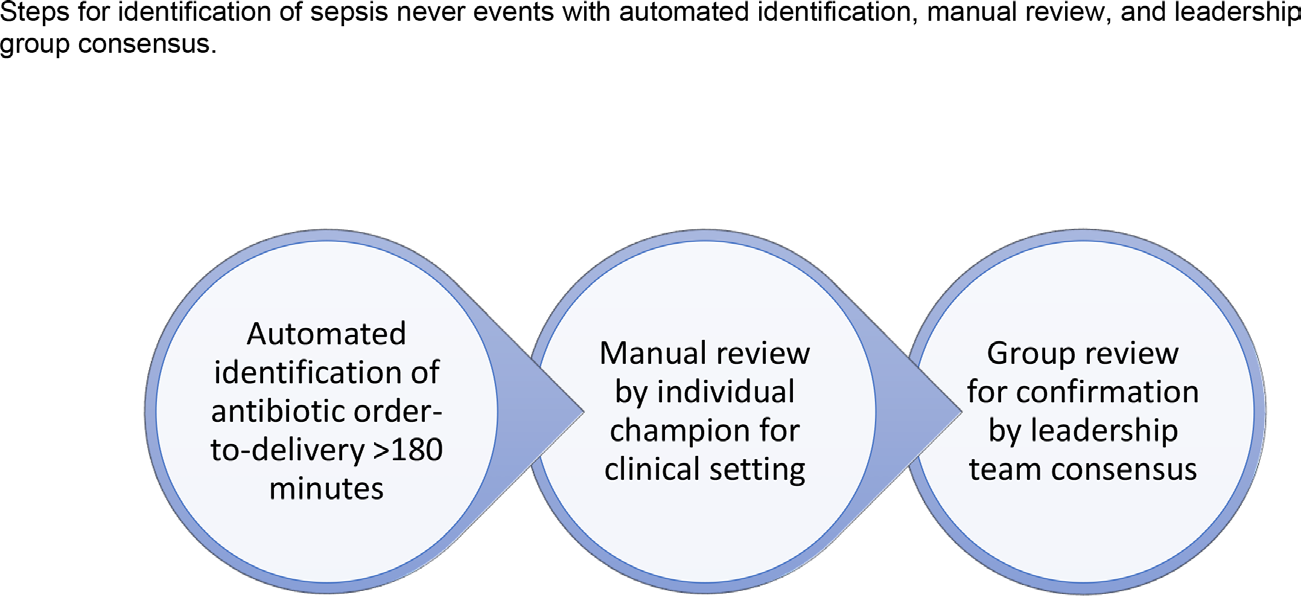
Steps for identification of Sepsis Never Events with automated identification, manual review, and leadership group consensus.

Our sepsis leadership team reviewed existing sepsis measures. Through iterative review and consensus, we defined a measure prioritizing final diagnosis rather than intent-to-treat. We created an automated identification and manual chart review process to identify cases meeting criteria (Figs. 1, 2). Cases meeting criteria trigger in-depth case reviews in partnership with system-wide patient safety teams and unit leaders.

## Results:

Sepsis Never Events are confirmed sepsis cases with infection-related organ dysfunction, antibiotic order-to-delivery >180 minutes, and subject matter consensus that antibiotic delay led to patient harm.

## Conclusions:

Our novel measure targets zero harm for patients with sepsis and identifies deviant cases to facilitate in-depth review and continuous improvement.

